# Alectinib, an Anaplastic Lymphoma Kinase Inhibitor, Abolishes ALK Activity and Growth in ALK-Positive Neuroblastoma Cells

**DOI:** 10.3389/fonc.2019.00579

**Published:** 2019-07-05

**Authors:** Muhammad Wasi Alam, Marcus Borenäs, Dan E. Lind, Diana Cervantes-Madrid, Ganesh Umapathy, Ruth H. Palmer, Bengt Hallberg

**Affiliations:** Department of Medical Biochemistry and Cell Biology, Sahlgrenska Academy, University of Gothenburg, Gothenburg, Sweden

**Keywords:** neuroblastoma, alectinib, anaplastic lymphoma kinase (ALK), resistant mutations, xenograft, crizotinib, ALK inhibitors

## Abstract

Oncogenic receptor tyrosine kinases including anaplastic lymphoma kinase (ALK) are implicated in numerous solid and hematologic cancers. ALK mutations are reported in an estimated 9% of neuroblastoma and recent reports indicate that the percentage of ALK-positive cases increases in the relapsed patient population. Initial clinical trial results have shown that it is difficult to inhibit growth of ALK positive neuroblastoma with crizotinib, motivating investigation of next generation ALK inhibitors with higher affinity for ALK. Here, alectinib, a potent next generation ALK inhibitor with antitumor activity was investigated in ALK-driven neuroblastoma models. Employing neuroblastoma cell lines and mouse xenografts we show a clear and efficient inhibition of ALK activity by alectinib. Inhibition of ALK activity was observed *in vitro* employing a set of different constitutively active ALK variants in biochemical assays. The results suggest that alectinib is an effective inhibitor of ALK kinase activity in ALK addicted neuroblastoma and should be considered as a potential future therapeutic option for ALK-positive neuroblastoma patients alone or in combination with other treatments.

## Introduction

Anaplastic lymphoma kinase (ALK) was initially described as a fusion partner of nucleophosmin (NPM) in the NPM-ALK translocation in a cell line derived from a patient with anaplastic large cell lymphoma (ALCL) (1). The full length ALK receptor tyrosine kinase (RTK) (2, 3), is activated by ALKAL ligands (4–7). Numerous ALK fusion proteins have been described in a wide range of cancers including non-small-cell-lung-cancer (NSCLC), inflammatory immunofibroblastic tumors (IMT), and diffuse B cell lymphoma (DBCL) (8). In addition to ALK fusions, mutation of ALK in pediatric neuroblastoma has been reported (9–13). Neuroblastoma is a heterogeneous disease which displays common genomic aberrations, such as deletion of parts of chromosome arms 1p and 11q, gain of 17q (14, 15), and amplification of the MYCN oncogene (16, 17). ALK mutations occur in 9% of primary tumors but this percentage is increased in the relapsed neuroblastoma patient population (18–20). These are mostly point mutations in the kinase domain of the full-length ALK receptor, although deletions have also been described in the extracellular domain that constitutively activate ALK kinase activity (16, 21–23).

Over the last decade a number of ALK tyrosine kinase inhibitors (TKIs) have been developed and approved for treatment of patients with ALK-positive NSCLC (24). The first generation drug crizotinib displayed antitumor activity against ALK and c-Met in experimental models of ALCL (25). In the initial phase I trial crizotinib demonstrated an impressive objective response rate (26). A subsequent trial that randomized chemotherapy-naïve patients with advanced ALK-positive NSCLC to receive crizotinib or chemotherapy showed longer progression free survival in the crizotinib arm compared to chemotherapy, although no difference in overall survival was observed (27). Although responses to ALK TKIs are very positive initially, the majority of ALK-positive patients develop an acquired resistance leading to complex treatment strategies and disease monitoring (28–30). Since crizotinib, a number of next generation ALK inhibitors have been approved and/or are included in late phase III clinical trials for NSCLC ALK fusion patients (30). These ALK TKIs differ in their ability to inhibit different ALK mutations, since certain mutations mediate steric hindrance that impair binding of a particular drug to the kinase (24, 29).

Alectinib is a next generation orally available, highly selective, ATP-competitive ALK TKI (31–34), that was approved in July 2014 (Japan) and December 2015 (US) to treat relapsed ALK-positive NSCLC as well as patients that were unable to tolerate crizotinib. Alectinib has improved ALK inhibition and blood brain barrier transport when compared with crizotinib and reports show an longer median progression free survival (35–37) as well as an improved safety profile in NSCLC compared with crizotinib (36, 38). Moreover, alectinib exhibits strong activity toward the EML4-ALK-L1196M gatekeeper and the ALK-F1174L and ALK-R1275Q mutations (31).

Here we investigate the pre-clinical potential of alectinib in an ALK positive neuroblastoma context. We have examined the ability of alectinib to abrogate the activity of different full length ALK gain-of-function mutations found in neuroblastoma cases, looking at neurite outgrowth, cell cycle progression and induction of apoptosis. Mouse xenografts were employed to investigate the ability of alectinib to inhibit tumor growth in a mouse neuroblastoma model. We show that alectinib inhibits growth in neuroblastoma cell lines, cells expressing neuroblastoma specific ALK mutations and also in exogenously expressed ALK activity in mouse model xenografts. Taken together, our results indicate that alectinib is an effective inhibitor in an ALK-positive neuroblastoma setting.

## Materials and Methods

### Reagents, Cell Lines and Antibodies

Alectinib (#S2762) and crizotinib (#S1068) were purchased from Selleckchem. The neuroblastoma cell lines, CLB-BAR (amplified MYCN/ALK, ALK^Δ*exon*4−11^), CLB-GE (amplified MYCN/ALK, ALK^F1174V^), SK-N-AS (11q-del, K-Ras^Q61K^), CLB-PE (amplified MYCN, ALK-wt), and IMR-32 (amplified MYCN, ALK-wt) and PC12 cells were cultivated and grown as reported previously (39, 40). For immunoblotting, phospho-ALK (Y1604) (Rabbit, 1:1,000, Cell Signaling Technology, #3341L), N-MYC (Rabbit, 1:1,000, Cell Signaling Technology, #9405S), phospho-AKT (S473) (Rabbit, 1:1,000, Cell Signaling Technology, #4060L), AKT (Rabbit, 1:1,000, Cell Signaling Technology, #9272), phospho-ERK1/2 (Rabbit, 1:2,000, Cell Signaling Technology, #4377S), pan-ERK (Mouse, 1:5,000, BD Biosciences, #610124) and actin (Rabbit, 1:5,000, Cell Signaling Technology, #4970S) from Cell Signaling Technology (Denvers, MA), were used as primary antibodies. Anti-ALK, monoclonal antibody 135 (Mouse, 1:5,000) was described (41, 42). Secondary antibodies were goat anti-rabbit and anti-mouse (1:5,000), purchased from Thermo Fisher Scientific. Reagents used in tumor immunohistochemistry were anti-CD31 (Rabbit, 1:500, Cell Signaling Technology, #77699S), Cleaved Caspase 3 (Rabbit, 1:500, Cell Signaling Technology, #9661S), Ki-67 (Rabbit, 1:400, Cell Signaling Technology, #9027), anti-phospho-Histone H3 (Ser10) (Rabbit, 1:500, Millipore, 06-570) as well as Signalstain®antibody diluent, Signalstain®Boost IHC detection reagent, Signalstain®DAB chromogent diluent and Signalstain®DAB chromogen were obtained from Cell Signaling Technology. Antibody against phospho-Histone H3 (Ser10) (Rabbit, 1:500, Millipore, #06-570) was obtained from Millipore. Normal goat serum was purchased from Jackson ImmunoResearch Laboratory.

### Cell Culture and Lysis

RPMI-1640 medium was used to culture neuroblastoma cell lines, supplemented with 10% fetal bovine serum (FBS) and 1% penicillin/streptomycin, PC12 cells were cultured in MEM medium with 7% horse serum, 3% FBS, and 1% penicillin/streptomycin at 37°C, 5% CO_2_, 95% humidity. CLB-BAR and CLB-GE cells were plated into 6-well plates (1 × 10^6^ cells/well). Alectinib (0 nM to 125 nM) was added to starvation medium (RPMI-1640 without FBS) to cells for 6 h. Cold PBS was used to wash the cells and cells were harvested in lysis buffer [25 mM Tris-Cl, pH 7.5, 150 mM of NaCl, 1% (v/v) Triton X-100, 1 mM DTT and protease inhibitor cocktail tablet (Roche)]. Cell lysates were collected after centrifuging at 14,000 rpm for 15 min at 4°C, SDS sample buffer was added to the lysate, followed by boiling and analyzed by immunoblotting as described previously (42).

### PC12 Cell Culturing and IC_50_ Determination

Transient transfection was performed as described previously with various ALK constructs (42) in PC12 cells (3 × 10^6^ cells/transfection), by electroporation in an Amaxa electroporator using 0.75 μg of the specified ALK mutant variants (1.5 μg of ALK-wt was used) and 100 μl of Ingenio electroporation solution (Mirrus Bio LCC). Alectinib and crizotinib were added at the specified concentrations (0–1,000 nM) for 4 h, washed with PBS and lysed with 1 × sample buffer (prepared from 4 × sample buffer−3% SDS, 100 mM Tris (pH 6.8), 100 mM DDT, 50 mM EDTA, and 40% glycerol). Samples were analyzed by immunoblotting after boiling at 95°C for 5 min, as described (39, 43). Image studio lite 3.1 was used to quantify phosphor-ALK protein and normalize to control sample (0 nM inhibitor samples). The IC_50_ were analyzed as log (inhibitor) vs. normalized response using GraphPad Prism 7.0. Data is presented as ±SD. Three independent experiments were performed.

### Cell Proliferation

2 × 10^4^ cells were seeded in 48 well plates with the indicated various concentration of either alectinib (0, 10, 20, 40, 80, 160 and 320 nM) or crizotinib (0, 10, 20, 40, 80, 160 and 320 nM). Cell viability was analyzed by adding resazurin (Sigma, Sweden) after 72 h incubation. IC_50_ values were generated by using GraphPad Prism 7.0. Each experiment was repeated at least three times and performed in triplicate. Further, neuroblastoma cell lines were also seeded into 48-well plates to achieve 30-40% confluency at the time of treatment. The cells were placed in an IncuCyte S3 (Essen BioScience, MI, USA) for 4 days, the first time point corresponds to cells prior to treatment, after the first image acquisition the cells were treated with the different drugs and placed again in the IncuCyte S3. For each drug, six different concentration were tested; alectinib (0, 40, 80, 160, 320, and 640 nM) and crizotinib (0, 100, 200, 300, 400, and 500 nM). Sixteen images per well were taken using the 10 × magnification objective for the phase contrast channel. All the images were processed and analyzed using the IncuCyte S3 live-cell imaging software. The analysis definition was created as a basic analyzer, phase contrast channel and selecting 6–8 representative images to test the mask. The segmentation and the minimum area (μm^2^) filters were adjusted to achieve a maximum detection of cells excluding debris. The analysis definition was done for each cell line separately and those specific parameters were used for all the images in each cell line group. Each experiment was repeated three independent times and performed in triplicate. IC_50_ values were calculated using log (inhibitor) vs. normalized response in GraphPad Prism 7.0.

### Cell Cycle Analysis

Neuroblastoma cells were seeded in 6-well plates (1 × 10^6^ cells per well) pre-coated with 0.4% solution of Type I Bovine collagen solution (Advance BioMatrix, LOT#7434). Cells were treated with alectinib (100 nM) for 24 h. Cells were fixed and treated according to the fixed cell cycle-DAPI assay protocol (NucleoCounter NC-3000, Chemometec, Denmark), and cell cycle was determined using the NucleoCounter NC-3000. Analysis of the data was done using the Nucleoview NC-3000 (Chemometec, Denmark).

### Neurite Outgrowth

PC12 cells (2 × 10^6^ cells) were transfected in 100 μl of Ingenio electroporation solution (Mirrus Bio LCC) with pENGFPN1 (Clonetech) (0.5 μg) and ALK-wt or ALK constructs (0.75 μg), prior to plating in 24-well plates. Transfected cells were treated with different concentrations of inhibitors for 48 h. For calculation, the fraction of neurite carrying GFP-positive cells vs. GFP-positive cells was analyzed. Cells that had a neurite with double the length of a normal cell were considered as neurite outgrowing cells and these cells were identified employing a Zeiss Axiovert 40 CFL microscope.

### Mouse Model

Female BALB/cAnNRj-Foxn1nu mice (Janvier Laboratory) at 5–6 weeks of age were subcutaneously injected with 1 × 10^6^ CLB-BAR cells in serum-free medium mixed at a ratio of 1:1 with Matrigel Matrix (lot. #6140322, Corning), in a total injection volume of 100 μl, into the left flank. Once tumor volume reached an average of 150 mm^3^, the mice were randomized to four treatment groups, vehicle (*n* = 10), alectinib (*n* = 10), crizotinib (*n* = 10), and repotrectinib (*n* = 10). Results for repotrectinib will be presented elsewhere. Alectinib and crizotinib were administered at 20 mg/kg and 80 mg/kg bodyweight, respectively, once daily continuously for 14 days. Tumor volume was measured by calipers every second day and calculated by the following equation: V = (π/6) × L × W^2^ (V, volume; L, longest; W, width). The vehicle for all compounds was 1% Carboxymethylcellulose sodium salt (21902, Sigma-Aldrich, Lot # BCBN1690V), 0.5% Tween-80 (P1754, Sigma-Aldrich, Lot # BCBT0817).

### Tumor Immunohistochemistry

At the end of the experiment xenograft tumors (*n* = 5 for each tumor category) were harvested and fixed in 4% paraformaldehyde for 72 h. Following fixation, the tumors were imbedded in paraffin blocks and sectioned in 5 μM slices with a manual microtome. Heat-induced epitope retrieval (HIER), using citrate buffer 0.01 M, pH 6, was performed before staining. HIER was achieved through a sequence where citrate buffer, containing the slides, was brought up to boiling, sub-boiled for 5 min following 10 s of intermediate cooling. The sequence was performed three times with cooling (5 min) in between. Following washing in distillated H_2_O (3 × 5 min), the slides were immerged in 3% H_2_O_2_ for 15 min and then washed in tris-buffered saline-Tween 20 (TBST) for 5 min. A hydrophobic pen was used to set a margin encircling the samples on the slides. Blocking was achieved by diluting normal goat serum (Jackson ImmunoResearch Laboratory, 005-000-121) in TBST to a concentration of 5%, adding the mixture to the slides followed by incubation in RT for 1 h. Antibodies were prepared by dilution in Signalstain® antibody diluent (Cell Signaling Technology, #8112S): anti-Ki-67 (Rabbit, 1:400, Cell Signaling Technology, #9027), anti-phospho-Histone H3 (Ser10) (Rabbit, 1:500, Millipore, 06-570), anti-Cleaved caspase 3 (Rabbit, 1:500, Cell Signaling Technology, #9661S), anti-CD31 (Rabbit, 1:500, Cell Signaling Technology, #77699S). The slides were incubated for 48 h in a cold room after being covered with antibody diluent. The slides were washed in TBST (3 × 5 min) and then covered in Signalstain® Boost IHC detection reagent (HRP, Rabbit, Cell Signaling Technology, #8114S) for 30 min in RT. Additional washing steps in TBST (3 × 5 min) were carried out. A mixture of Signalstain® DAB chromogen and DAB diluent (Cell Signaling Technology, #8059S) was used according to the manufactures instructions. The slides were counterstained with Mayer's hematoxylin solution (Sigma-Aldrich SLBK8961V), dehydrated and mounted.

### Image Acquisition and Quantification

Hamamatsu NanoZoomer-SQ Digital slide scanner (C13140-01) with a x20 (NA 0.75) objective was used to obtain digital images of the slides. Slides were randomly blinded to the investigator. For each of the blinded slides, a representative 1 mm^2^ area was selected employing NanoZoomer Digital Pathology viewer. The slide-image was cropped, containing the area of interest, and saved, as a TIF-file at × 20 resolution. The saved TIF-files were cropped, using ImageJ (Fiji) (44), into merely encompassing the 1 mm^2^ area of interest.

### Quantification of Immunohistochemistry

The 6–7 images were then uploaded into Ilastik (45), an interactive machine-learning toolkit, and used as a learning foundation for the software (see program code, Supplementary Data Sheet 3). Once the software analyzed the learning images, the whole batches were processed in Ilastik. The output was then transferred to ImageJ where a macro (see program code, Supplementary Data Sheet 2) calculated the area of staining. The pixel size acquired from NanoZoomer Digital Pathology viewer was accounted for in the macro. Ki-67 immunohistochemistry was also analyzed manually. Briefly, five representative sample areas from each treatment arm (alectinib, crizotinib, and vehicle treated animals) were analyzed blindly by 4 independent investigators. The mean manual counts from alectinib or crizotinib treated tumors were normalized against vehicle. The differences in median between the treated groups and vehicle were calculated in Microsoft Exel 2016. Ninety five percent confidence intervals for the difference between median were retrieved with GraphPad.

### Statistical Analysis of Tumor Immunohistochemistry

GraphPad Prism 7.0 was used for statistical analysis. A non-parametric test was performed acquiring *P*-values by applying a Mann-Whitney test.

## Results

### Alectinib Inhibits ALK Activity and Proliferation of ALK Addicted Neuroblastoma Cell Lines

**Figure 1:**
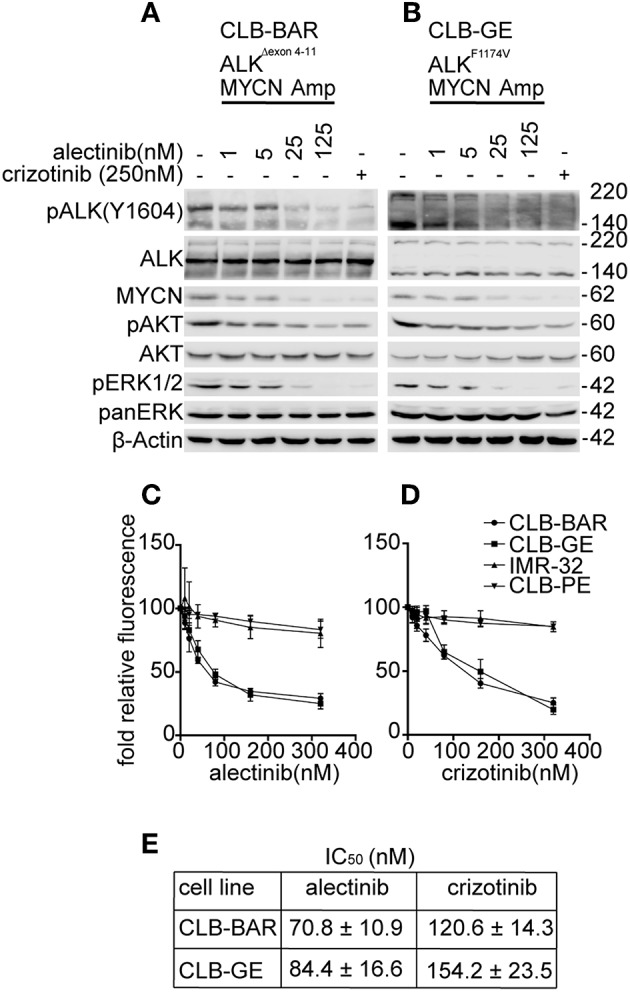
Alectinib inhibits signaling in ALK addicted neuroblastoma cell lines. **(A,B)** ALK addicted cell lines, CLB-BAR (amplified MYCN/ALK, ALKΔexon 4-11), and CLB-GE (amplified MYCN/ALK, ALK-F1174V) were treated with alectinib at the concentrations indicated for 6 h and lysate immunoblotted for pALK-Y1604 and the ALK downstream targets ERK1/2, AKT, and MYCN. Crizotinib (250 nM) was used as a positive control. ALK runs at approximately 170 kDa in CLB-BAR cells due to a genomic deletion in ALK between exon 4-11 (22, 48). In CLB-GE cells, the mutant full-length ALK-F1174V mutant is cleaved and is detected as two bands. **(C,D)** CLB-PE and IMR-32 harbor a wildtype ALK and are ALK-wt and non-ALK addicted neuroblastoma cell lines. All four neuroblastoma cell lines were grown in increasing concentrations of either alectinib **(C)** or crizotinib **(D)** for 72 h after which cell viability was monitored by resazurin assay (Sigma, Sweden). Mean ± SD values were plotted from three independent experiments, performed in triplicate. **(E)** IC_50_ values from the cell viability analysis in C and D were generated in GraphPad Prism 7.0. The results were analyzed as log (inhibitor) vs. normalized response and are expressed as mean ± SD.

A panel of neuroblastoma cell lines, including CLB-BAR (amplified MYCN/ALK, ALKΔexon4-11), CLB-GE (amplified MYCN/ALK, ALK-F1174V), CLB-PE (amplified MYCN), and IMR-32 (amplified MYCN) was used to test alectinib (39, 40, 46, 47). We have previously shown that the two ALK positive cell lines, CLB-BAR and CLB-GE, are dependent on ALK activity for growth, while IMR-32 and CLB-PE are not (39, 40, 46, 47). The effect of alectinib treatment on ALK signaling was analyzed by immunoblotting (Figures 1A,B). Crizotinib (250 nM) was included as a positive control. Alectinib treatment of CLB-BAR and CLB-GE cell lines resulted in reduction of ALK activity as measured by pALK-Y1604 in a dose-dependent manner (Figures 1A,B). Crizotinib showed inhibition of pALK-Y1604 at 250 nM (Figures 1A,B), consistent with earlier reports (39, 40, 46, 47). Along with the reduction of pALK-Y1604, phosphorylation of the downstream signaling targets AKT and ERK1/2 was also decreased. In addition, MYCN protein levels decreased in the presence of either alectinib or the crizotinib positive control (Figures 1A,B).

The proliferation of both CLB-BAR and CLB-GE was more sensitive to alectinib treatment as compared to crizotinib (Figures 1C,D). The IC_50_ for alectinib in CLB-BAR and CLB-GE was 70.8 ± 10.9 and 84.4 ± 16.6, respectively, while for crizotinib the values for CLB-BAR and CLB-GE were 120.6 ± 14.3 and 154.2 ± 23.5, respectively (Figure 1E). Analysis of cell proliferation was also performed with an Incucyte live cell analysis system with similar results (Supplementary Figure 1). Neither alectinib nor crizotinib were able to inhibit growth of non-ALK addicted CLB-PE and IMR-32 neuroblastoma cell lines (Figures 1C,D), suggesting that neither alectinib nor crizotinib was toxic at the levels employed. Thus, alectinib blocks ALK signaling and pathway activity more effectively when compared with crizotinib and with no obvious toxic effects.

### Alectinib Inhibits Constitutively Active ALK Mutants

**Figure 2:**
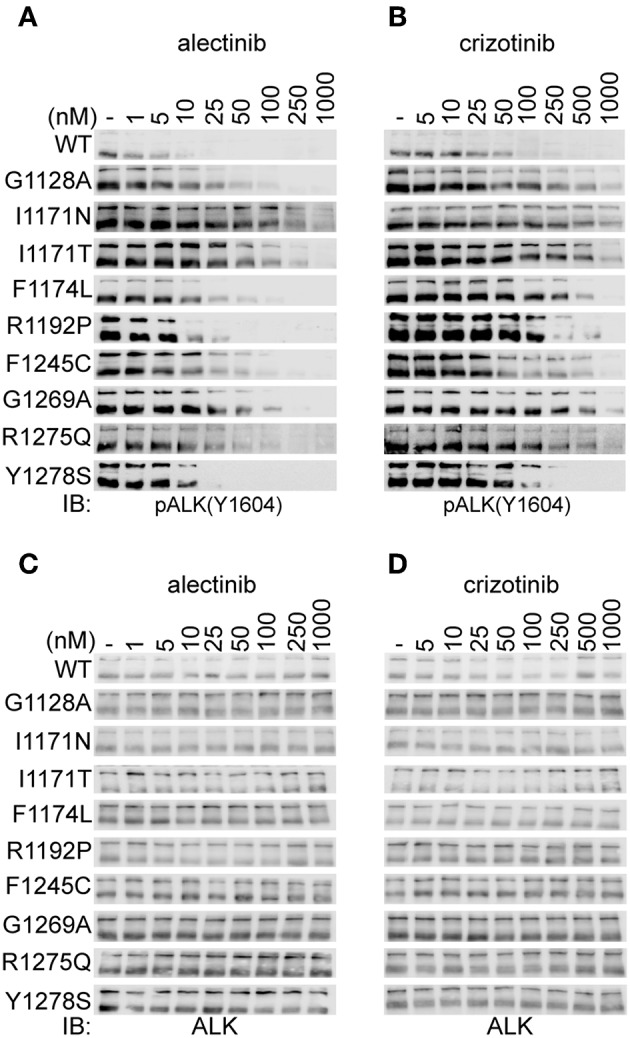
Alectinib inhibits activity of ALK mutants in PC12 cells. **(A,B)** PC12 cells transiently transfected with either ALK-wt (1.5 μg) or ALK mutant variants as indicated (0.75 μg), were treated with increasing concentrations of either alectinib **(A)** or crizotinib **(B)** at 48 h post-transfection. ALK activation was measured by immunoblotting for pALK-Y1604. **(C,D)** Shows the total ALK levels (as a loading control) for **(A,B)**, respectively.

To extend our analysis, we examined the ability of alectinib to inhibit ALK gain of function variants found in neuroblastoma. Known gain of function ALK mutations ALK-G1128A, ALK-I1171N, ALK-I1171T, ALK-F1174L, ALK-R1192P, ALK-F1245C, ALK-R1275Q, and ALK-Y1278S were transiently expressed in PC12 cells. We also included ALK-G1269A, which represents a resistant mutation reported in an EML4-ALK fusion protein in a non-small-cell-lung-cancer cases. Inhibition of ALK phosphorylation was measured and estimated by immunoblotting for pALK-Y1604 upon treatment with alectinib and crizotinib in a dose-dependent manner (Figures 2A,B). The ALK loading control blots are included as Figures 2C,D. The ALK levels of all mutants at same time point and drug concentration are shown in Supplementary Figure 2.

ALK-wt and the ALK-G1128A, ALK-F1174L, ALK-F1245C, and ALK-R1275Q mutant variants show similar sensitivity to crizotinib as has been previously reported (39, 40, 46, 47). However, ALK-I1171N, ALK-I1171T, and ALK-G1269A mutant variants required a higher dose of crizotinib for efficient inhibition of phosphorylation of ALK-Y1604 as has been previously reported (Figures 2A,B and Table 1) (46, 47). Alectinib inhibited the activation of ALK gain of function alleles with an IC_50_ level that was 5 to 22-fold lower than crizotinib with one exception, ALK-I1171N (Figures 2A,B and Table 1). Generally, alectinib abrogated activity of ALK mutant variants with an IC_50_ level of single digit nM with the exception of the ALK-I1171T and ALK-I1171N mutations which were 28.4 ± 2.1 and 52.5 ± 19.1, respectively. Our investigation of ALK gain of function mutation variants suggested an effective inhibition profile for alectinib as compared with crizotinib for all constitutively active neuroblastoma mutations with the exception of mutations at ALK-I1171.

**Table 1:** IC_50_ values of pALK-Y1604 inhibition by either alectinib or crizotinib treatment after 4 h in PC12 cells for ALK-wt and the indicated ALK neuroblastoma mutant variants.

	**IC**_**50**_ **(nM)**		**Fold difference**
**hALK mutations**	**Alectinib**	**Crizotinib**	
WT	1.7 ± 0.4	29.0 ± 3.1	17
G1128A	11.1 ± 1	56.0 ± 12.7	5
I1171N	52.5 ± 19	117.1 ± 32.7	2.2
I1171T	28.4 ± 2.1	130 ± 27.7	4.5
F1174L	3.5 ± 0.3	31.6 ± 4.8	9
R1192P	4.3 ± 0.7	58.9 ± 2.6	14
F1245C	3.6 ± 0.6	46.5 ± 1.5	13
G1269A	14.7 ± 0.8	166.6 ± 17.1	11
R1275Q	2.8 ± 0.1	33.0 ± 2.0	12
Y1278S	3.0 ± 0.1	66.4 ± 0.6	22

*Quantification of pALK-Y1604 was performed from the experimental analysis shown in Figures 2A,B. Three independent experiments were performed. IC_50_ quantification was analyzed by normalizing pALK (Y1604) against panERK (loading control). The quantified values were analyzed as log (inhibitor) vs. normalized response using GraphPad Prism 7.0, and are expressed as mean ± SD. The fold difference indicates as fold relative to crizotinib*.

### Alectinib Promotes Cell Cycle Arrest and Induction in Apoptosis

**Figure 3:**
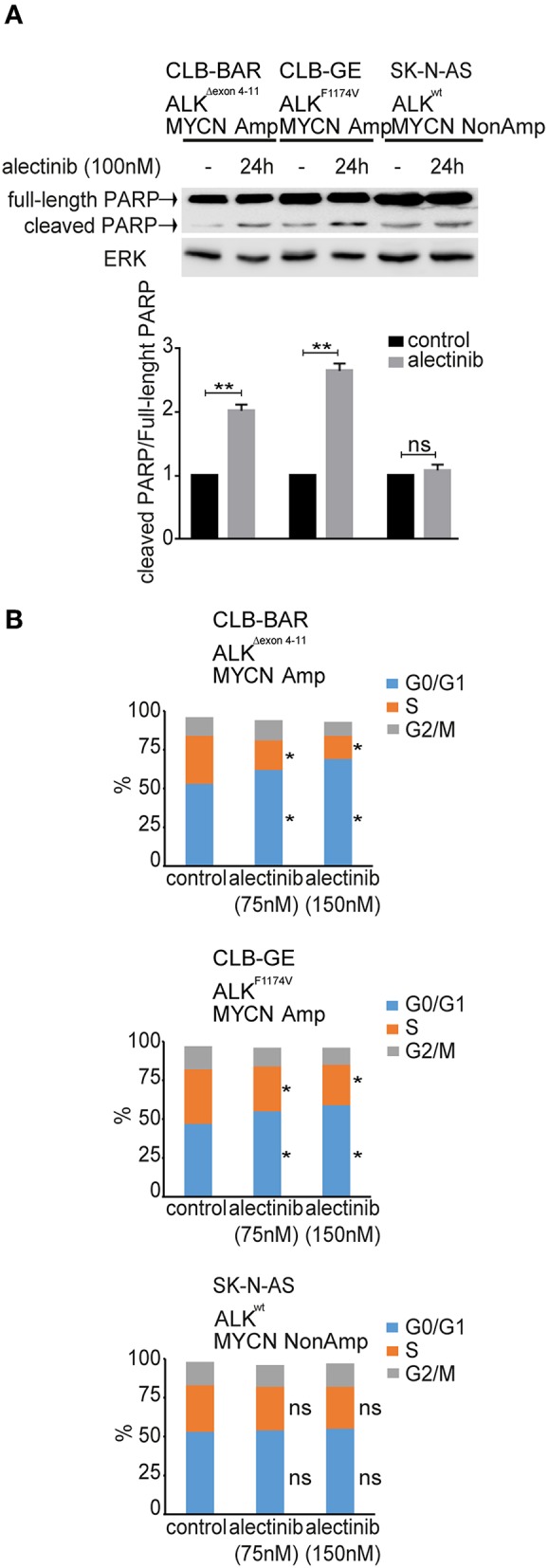
Cell cycle analysis and apoptosis analysis upon treatment of neuroblastoma cells with alectinib. **(A)** CLB-BAR, CLB-GE, and SK-N-AS cells were grown on six-well plates with complete growth medium and treated with alectinib (100 nM) for 24 h. Cell lysates were immunoblotted with antibodies against PARP. ERK was used as a loading control. Three independent experiments were performed (biological replicates). Values were analyzed by normalizing, the fraction of full-length PARP vs. cleaved PARP and calculated in GraphPad Prism 7.0, and are expressed as mean ± SD. *P*-values were calculated by Student paired *t*-test, ^**^p-value > 0.005; ns, non-significant. **(B)** Cell cycle analysis of CLB-BAR, CLB-GE, and SK-N-AS cells treated with alectinib 75 and 150 nM for 24 h. The data was normalized to the respective control. Three independent experiments were performed (biological replicates). Control vs. alectinib (75 nM) and control vs. alectinib (150 nM) in G0/G1 phase (blue bar) and S phase (orange bar) showed significant difference in both CLB-BAR and CLB-GE, but not in SK-N-AS. *P*-values were calculated by Student paired *t*-test, ^*^p-value <0.05; ns, non-significant.

To investigate the response of cells to alectinib treatment we performed a cell cycle and apoptosis analysis. We observed a significant increase of cleaved PARP levels in ALK dependent neuroblastoma cell lines, such as CLB-BAR and CLB-GE in response to alectinib treatment, while no difference was observed in SK-N-AS (11q-del, K-Ras^Q61K^), CLB-PE and IMR-32 control cell lines (Figure 3A and Supplementary Figure 3). Both CLB-BAR and CLB-GE ALK positive cells showed inhibition of cell cycle progression after 24 h ALK TKI treatment, which resulted in increased G1 accumulation that was not observed in the SK-N-AS control cell line (Figure 3B). Our observations indicate that alectinib treatment results in delayed cell cycle progression in ALK addicted cells together with an induction of apoptosis.

### Alectinib Blocks ALK Activation and ALK-Mediated Neurite Outgrowth

**Figure 4:**
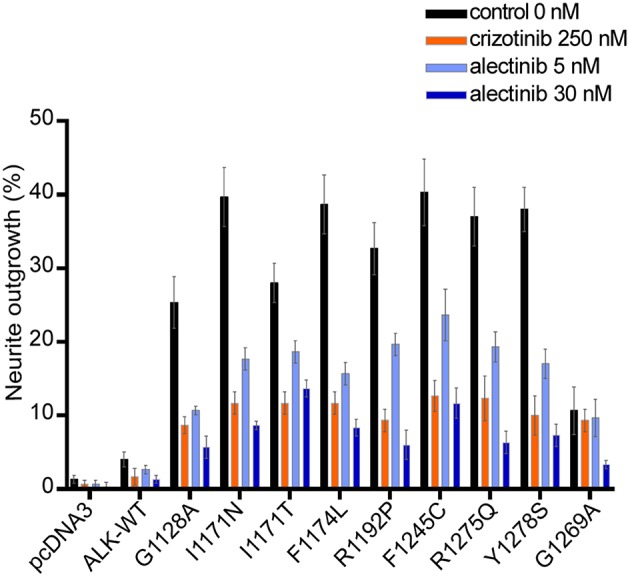
Alectinib inhibits ALK mediated neurite outgrowth efficiently in PC12 cells. ALK mutant variants were co-transfected with pEGFPN1 in PC12 cells. Neurite outgrowth was measured in GFP-positive cells 48 h post-treatment with either alectinib or crizotinib at the specified concentration. Those GFP cells having neurite outgrowth double the size of cells were determined as a positive neurite-carrying cells. Data shows the percentage of GFP neurites-carrying cells Graph shows the mean of three independent experiment ± SD.

We next investigated the ability of alectinib to inhibit ALK driven PC12 cell neurite outgrowth. PC12 cells are clonal rat adrenal pheochromocytoma cell line with enteric cell origin, that differentiate and spread neurites in response to stimulation (49). We and others have previously reported that activation of ALK triggers differentiation of PC12 cells, a process that is characterized by extension of neurites (39, 42, 46, 47, 50). Neurite outgrowth was abrogated by both alectinib and crizotinib (Figure 4). Inhibition of neurite outgrowth by wildtype ALK was observed at low concentrations of alectinib treatment (5 nM), and was more effective at 30 nM. While inhibition of some ALK mutant variants, e.g., I1171N/T, was less sensitive to 5 nM alectinib (Figure 2A) inhibition for longer time was able to inhibit these variants in neurite outgrowth assays. In agreement with our earlier observations alectinib displayed increased efficacy when compared with crizotinib, with 30 nM alectinib resulting in a similar or greater reduction of neurite outgrowth than 250 nM crizotinib.

### Alectinib Shows Robust Anti-tumor Growth in a Xenograft Model of Neuroblastoma

**Figure 5:**
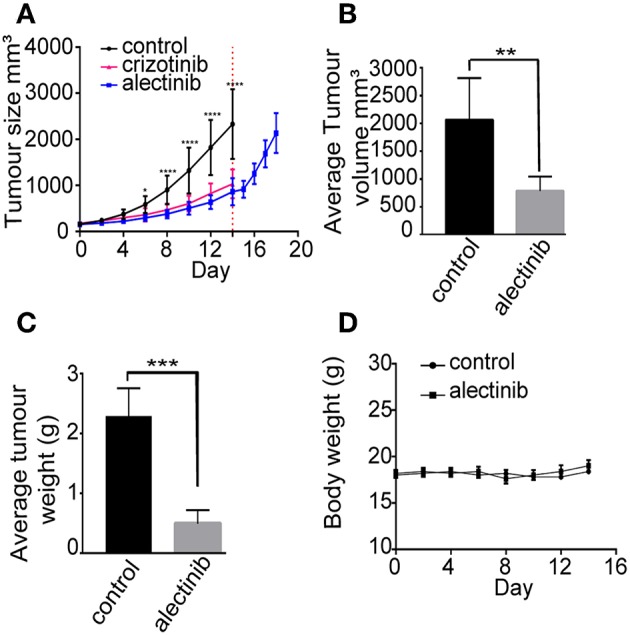
Alectinib inhibits tumor growth in a neuroblastoma xenograft model. 1 × 10^6^ CLB-BAR cells were injected subcutaneously in female BALB/cAnNRj-Foxn1nu mice. Mice were treated with vehicle control (*n* = 10), crizotinib (80 mg/kg once daily, *n* = 10) or alectinib (20 mg/kg once daily, *n* = 10) upon tumor growth. **(A)** Curves indicate tumor volume with vehicle control, crizotinib or alectinib treatment over 14 days. Red dotted line indicates the last day of treatment (day 14). The samples after red dotted line indicate the tumor growth after release from alectinib treatment (*n* = 5). Values were calculated with a two-way ANOVA, with Sidak's multiple comparison test employing GraphPad Prism 7.0, ^*^p = 0.0222 and ^****^p < 0.0001. **(B)** Average tumor volume in either control or alectinib treated groups after 14 days (*n* = 5). **(C)** Average tumor weight in either control (*n* = 5) or alectinib treated groups after 14 days (*n* = 5). **(D)** Body weight of either control or alectinib treated mice during treatment. **(B,C)** values were calculated in GraphPad Prism 7.0, and are expressed as mean ± SD, *P*-values were calculated with an unpaired two-tailed Student *t*-test, ^***^p-value < 0.0001 **(C)** and ^**^p-value = 0.0072 **(B)**.

Next generation ALK TKIs such as lorlatinib and brigatinib have been shown to be more effective than crizotinib as single agent neuroblastoma xenografts (46, 47). In order to investigate the potency of alectinib as a single agent in a mouse neuroblastoma model, CLB-BAR cells were injected subcutaneously into BalbC/NUDE mice. Upon tumor growth, either alectinib (20 mg/kg bodyweight once daily), crizotinib (80 mg/kg bodyweight once daily) or vehicle (control) was administered continuously for 14 days. Alectinib effectively abrogated growth of neuroblastoma xenografts (Figure 5A). Xenograft tumor volume was significantly reduced already at day 6 (*p* = 0.0222) when compared with the vehicle treated groups and at day 8 (*p* < 0.0001). At the completion of alectinib treatment after 14 days reduction in tumor volume remained significant (*p* = 0.0072) (Figure 5B). At 14 days tumor weight was also significantly different between the alectinib treated group compared with the vehicle treated control group (*p* < 0.0001) (Figure 5C). Tumor growth was resumed when alectinib treatment was discontinued after 14 days (red dotted line), indicating that the observed inhibition on tumor growth was solely dependent on alectinib (Figure 5A). Treatment with alectinib was more effective than with crizotinib (Figure 5A). No difference in body weight or obvious side effects were observed during treatment with either control or alectinib treatment (Figure 5D).

**Figure 6:**
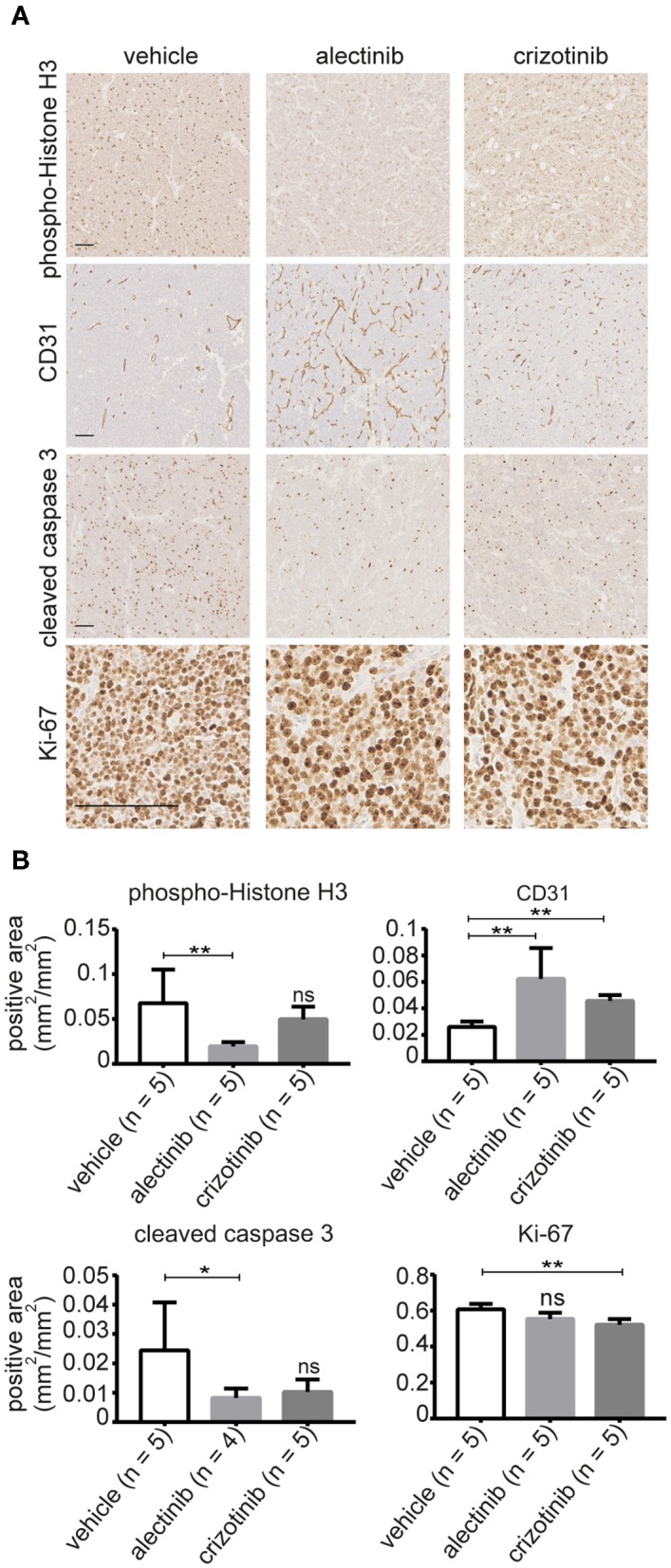
**(A)** Representative images of tumors treated with either alectinib, crizotinib, or vehicle and stained with anti-Ki-67, anti-CD31, phosphor-Histone H3, and cleaved caspase 3. **(B)** Bar graphs show mean values ± SD; *P*-values were calculated by Mann-Whitney test, ^**^p-value ≤ 0.008, ^*^p-value < 0.05; ns, non-significant. Scale bars indicate 100 μm.

To further investigate the effect of alectinib, tumors were removed from animals treated with alectinib, crizotinib or vehicle at day 14 for analysis. Ki-67 was used as a marker for proliferation along with phospho-histone H3, while CD31 and cleaved caspase 3 were used as markers for vascularization and apoptosis, respectively. Tumors treated with alectinib displayed a significant decrease in phospho-histone H3, indicating reduced mitosis, which was not observed in tumors treated with crizotinib (Figures 6A,B). This is in agreement with our cell cycle analysis (Figure 3B) which indicate that alectinib induces a G1-arrest. Analysis of Ki-67 levels by Ilastik resulted in a non-significant decrease in Ki-67 positivity in tumors treated with alectinib, while tumors treated with crizotinib showed a modest, but significant reduction in Ki-67 levels (Figures 6A,B). We noticed however that numbers of cells were different, with non-treated tumors displaying more cells per field. Therefore, Ki-67 levels were also subjected to manual analysis by 4 independent investigators. Alectinib showed a 0.46 (95% CI 0.32–0.52) lower median when compared to vehicle, while crizotinib displayed a 0.49 (95% CI 0.47–0.50) lower median when compared to vehicle. Alectinib treated tumors contained significantly lower numbers of cleaved caspase positive apoptotic cells at day 14 compared to vehicle, likely reflecting the decreased number of tumor cells per field at this time point, while crizotinib treated tumors showed a non-significant decrease in apoptosis (Figures 6A,B). Finally, CD31 was used as a marker for vascularization with a significant increase upon treatment observed with both alectinib and crizotinib (Figures 6A,B). Taken together, our results indicate that alectinib efficiently reduces xenograft tumor growth in a mono treatment scheme.

## Discussion

Activated ALK acts as an oncogenic driver in anaplastic large cell lymphoma (ALCL), non-small cell lung carcinoma (NSCLC) and neuroblastoma and is linked to poor clinical outcome, especially in high risk neuroblastoma (8, 16, 51). Targeted therapy has transformed the landscape of treatment options for ALK-positive cancers (35). Treatment with crizotinib in pediatric cancers such as NSCLC and IMT has shown an encouraging and efficient effect for tumors which harbor ALK fusion oncogenes (52), however, responses were less encouraging for treatment of pediatric neuroblastoma patients with crizotinib (53). Recently it was reported that mono treatment with ceritinib of a high-risk ALK neuroblastoma resulted in tumor shrinkage and the tumor was surgically removed. Clinical follow-up after 21 month treatment revealed complete clinical remission (54). The first clinical use of alectinib in a case of heavily pretreated, refractory metastatic ALK positive neuroblastoma was recently published (55). It is not known whether ALK mutation was present in the original tumor or developed at relapse, however, the patient remained stable for 16 weeks before onset of disease progression (55). Mutations in full length ALK found in neuroblastoma are observed within the kinase domain, and can influence the binding efficiency of the drug to inhibit activity and have implications for treatment choices and future drug combination strategies. The first report of alectinib focused mainly on inhibition of ALK fusion proteins, such as EML4-ALK and NPM-ALK (31). This study also showed that NB1 (ALK amplified) and Kelly (ALK-F1174L) neuroblastoma cells were sensitive to alectinib, and that ALK kinase domains harboring the F1174L and R1275Q mutations were sensitive to alectinib in *in vitro* kinase assays. These initial findings led us to perform an extensive analysis of different ALK mutant variants found in neuroblastoma in the context of the full-length receptor. Here we have investigated the ability of alectinib to abrogate the activity of ALK gain of function mutations found in neuroblastoma patients, examining this ALK TKI in ALK positive neuroblastoma cell lines, and in ALK driven xenografts.

Treatment with alectinib resulted in abrogation of growth and proliferation of cell lines harboring known ALK mutations. Previously described downstream targets such as ERK, AKT were less phosphorylated and MYCN expression levels were reduced upon treatment with alectinib. Control neuroblastoma cell lines were unaffected by alectinib and tolerated high doses, indicating low toxicity in the presence of drug. The most commonly found ALK mutations in neuroblastoma are ALK-F1174L, ALK-F1245C, and ALK-R1275Q (9–13). All three mutant variants were inhibited by alectinib in a preclinical setting in the low single digit nanomolar range (2.8 ± 0.1 to 3.6 ± 0.6 nM). Our results indicate a 9 to 13-fold difference between alectinib and crizotinib, indicating that alectinib is a more effective ALK TKI for these mutations. Alectinib abrogated all neuroblastoma gain of function mutations investigated here, including ALK-G1128A, ALK-R1192P, and ALK-Y1278S in a similar manner as the hot spot mutations. The ALK-I1171T and ALK-I1171N mutations show slightly higher IC_50_ values (28.4 ± 2.1 and 52.5 ± 19.1 nM, respectively) although alectinib is more effective at abrogating ALK activity compared with crizotinib. The increased resistance of the ALK-I1171 mutant variants to alectinib may be due to a steric hindrance between the ALK-I1171 mutants that impacts upon alectinib binding in the ATP-binding pocket of ALK. Isoleucine 1171 is an integral residue of the hydrophobic regulatory spine, which is composed of residues from both the N and the C-lobes of the kinase domain (56–58). It is important that the regulatory spine is correctly aligned for activity, and I1171 has been proposed to contribute to the internal dynamics of the kinase and mutation likely impacts the dynamic conformation of the ATP binding pocket (56–58). Alectinib also inhibits the ALK-G1269A mutation, which is a secondary mutation found in EML4-ALK positive crizotinib refractory NSCLC patients comparable well-compared with crizotinib and in the similar range as we have observed earlier with brigatinib (47). Neurite outgrowth of PC12 cells requires sustained activation of the Ras-MAPK pathway (49). Expression of constitutive activated ALK mutant variants drives neurite out-growth that is robustly inhibited by alectinib at low nM concentrations. Thus, alectinib exhibits strong activity toward many of the ALK neuroblastoma mutant variants with the exception of the ALK-I1171 mutations which are more resistant. The improved alectinib inhibition toward ALK activity compared with crizotinib is in the range of 5 to 20-fold based on our examination of the various constitutively active ALK mutant variants.

As has previously been shown, treatment with ALK TKIs inhibit activation of downstream targets of ALK, such as ERK1/2 and AKT, as well as MYCN expression. Here we observe that alectinib treatment reduces MYCN levels in a similar manner to that observed preciously with crizotinib, lorlatinib, brigatinib and ceritinib (42, 43, 46, 47, 54). Our results are in line with a recent report observing that alectinib inhibits cell proliferation in neuroblastoma cell lines (59). However, these authors reported suppression of cell proliferation by alectinib in both ALK positive cell lines and ALK negative cell lines with IC_50_ values of 3–10 μM (59). Here we show effective inhibition of proliferation of ALK dependent cell lines by alectinib at concentrations <100 nM. In addition to reduction of proliferation, we observe a 2 to 3-fold increase of the cleaved apoptosis marker PARP as well as an increased number of cells in the G0/G1 phase of the cell cycle on alectinib treatment in agreement with the findings of Miyazaki et al. (60) who reported that alectinib induces G1-arrest.

Employing a neuroblastoma xenograft mouse model we observed effective inhibition of tumor growth upon treatment with alectinib, indicating good potential for alectinib in tumor growth reduction *in vivo*. Taken together, our preclinical results show that alectinib is more efficient than crizotinib in the inhibition of ALK mutant variants observed in neuroblastoma patients. These data support the further investigation of alectinib as a therapeutic agent in ALK positive neuroblastoma.

## Ethics Statement

All experimental procedures performed were in accordance with the Regional Animal Ethics Committee approval (A230-2014), Göteborgs djurförsöksetiska nämnd, Jordbruksverket (Gothenburg Animal Ethics Committee, Swedish Board of Agriculture).

## Author Contributions

MA performed most of the cell and biochemical analyses together with DC-M and GU. MB and DL executed the mouse xenograft experiments and analyses. RP and BH designed the study and supervised the project. All authors were involved in further development and finalization of the manuscript.

### Conflict of Interest Statement

The authors declare that the research was conducted in the absence of any commercial or financial relationships that could be construed as a potential conflict of interest.
