# Supplementary material for: Alectinib, an Anaplastic Lymphoma Kinase Inhibitor, Abolishes ALK Activity and Growth in ALK-Positive Neuroblastoma Cells
Source: Front Oncol. 2019 Jul 5;9:579. doi: 10.3389/fonc.2019.00579 (PMC6625372; doi:10.3389/fonc.2019.00579)
Supplement: Supplementary file 1 [file Data_Sheet_1.docx]

Supplementary Material

**Supplementary Figure 1.** **IC_50_ values of neuroblastoma cells upon treatment with alectinib or crizotinib using IncuCyte S3.** Neuroblastoma cells were treated with different concentrations of alectinib or crizotinib for 3 days and cell proliferation was measured by an Incucyte. The results were analyzed as log (inhibitor) vs normalized response using GraphPad Prism 7.0. Data is presented as IC_50_ ± SD. The experiment was repeated three times in triplicate.

**Supplementary Figure 2.** **Expression of ALK mutants in PC12 cells.** Immunoblot analysis of total ALK for all ALK-mutant variants treated for 4h with 5nM alectinib. PanERK was employed as loading control.

**Supplementary Figure 3. Investigation of apoptosis in wild type neuroblastoma cell lines when treated with alectinib**. ALK-wt cell lines, CLB-PE and IMR-32, were treated with alectinib (100 nM) for 24h. Cell lysates were immunoblotted for PARP and ERK (employed as loading control). The bar graph shows the quantified values. Three independent experiments were performed. Values were calculated in GraphPad Prism 7.0, and are expressed as mean ± SD. P-values were calculated by student paired t-test, ns = non-significant).

**Supplementary Data Sheet 2. Program code for image processing**. Program code in ImageJ (version 1.51j8) for processing images developed by Hendrik Deschout from the Centre for Cellular Imaging at the University of Gothenburg and the National Microscopy Infrastructure, NMI (VR-RFI 2016-00968). Submitted with the permission of Centre for Cellular Imaging at the University of Gothenburg.

**Supplementary Data Sheet 3. Program code for cropping images**. Program code in ImageJ (version 1.51j8) for cropping images developed by Hendrik Deschout from the Centre for Cellular Imaging at the University of Gothenburg and the National Microscopy Infrastructure, NMI (VR-RFI 2016-00968). Submitted with the permission of Centre for Cellular Imaging at the University of Gothenburg.
