# Supplementary material for: Alectinib, an Anaplastic Lymphoma Kinase Inhibitor, Abolishes ALK Activity and Growth in ALK-Positive Neuroblastoma Cells
Source: Front Oncol. 2019 Jul 5;9:579. doi: 10.3389/fonc.2019.00579 (PMC6625372; doi:10.3389/fonc.2019.00579)
Supplement: Supplementary file 2 [file Data_Sheet_2.PDF]

```
// Measure areas corresponding to different labels as identified by Ilastik
// CCI - Hendrik Deschout - May 2018

// Ask user for folder that contains Ilastik output
dir = getDirectory("Choose a Directory");

// Get a list with all files
list = getFileList(dir);

// Define arrays in which to store the measured areas
negative_list = newArray(list.length);
positive_list = newArray(list.length);
background_list = newArray(list.length);

// Ask user for ROI width in nanometer and particle diameter in pixel
Dialog.create("User Input");
Dialog.addNumber("Pixel size (nm):", 444);
Dialog.show();
pixel_size = Dialog.getNumber() / 1000;

// Set counter
counter = 0;

// Loop through each file
for (i = 0; i < list.length; i++) {

        // Check if extension is "tif"
        if (endsWith(list[i], "tif")) {

                // Construct filepath
                path = dir + list[i];

                // Open file and get name
                open(path);
                title = getTitle();

                // Set measurements
                run("Set Measurements...", "area limit redirect=None decimal=3");

                // Measure the area for the background case
                selectWindow(title);
                run("Duplicate...", " ");
                setThreshold(1, 1);
                setOption("BlackBackground", true);
                run("Convert to Mask");
                run("Clear Results");
                run("Measure");
                background_list[counter] = getResult("Area", 0) * pixel_size *
pixel_size;
                close();

                // Measure the area for the negative case
                selectWindow(title);
                run("Duplicate...", " ");
                setThreshold(2, 2);
                setOption("BlackBackground", true);
                run("Convert to Mask");
                run("Clear Results");
                run("Measure");
                negative_list[counter] = getResult("Area", 0) * pixel_size * pixel_-
size;
                close();

                // Measure the area for the positive case
                selectWindow(title);
                run("Duplicate...", " ");
                setThreshold(3, 3);
                setOption("BlackBackground", true);
                run("Convert to Mask");
                run("Clear Results");
                run("Measure");
                positive_list[counter] = getResult("Area", 0) * pixel_size * pixel_-
size;
                close();

                // Close window and update counter
                close();
                counter++;

        }
}

// Clear Results table and set counter
run("Clear Results");
counter = 0;

// Loop through each file
for (i = 0; i < list.length; i++) {

        // Check if extension is "tif"
        if (endsWith(list[i], "tif")) {

                // Store the measured areas in the Results table
                setResult("file name", counter, list[i]);
                setResult("negative area (micron^2)", counter, negative_list[i]);
                setResult("positive area (micron^2)", counter, positive_list[i]);
                setResult("background area (micron^2)", counter, back-
ground_list[i]);
                setResult("positive-negative ratio", counter, positive_list[i] /
negative_list[i]);
                setResult("positive-total ratio", counter, positive_list[i] / (posi-
tive_list[i] + negative_list[i] + background_list[i]));
                setResult("negative-total ratio", counter, negative_list[i] / (posi-
tive_list[i] + negative_list[i] + background_list[i]));
                setResult("background-total ratio", counter, background_list[i] /
(positive_list[i] + negative_list[i] + background_list[i]));

                // Update counter
                counter++;

        }
}
```
