# Supplementary material for: Alectinib, an Anaplastic Lymphoma Kinase Inhibitor, Abolishes ALK Activity and Growth in ALK-Positive Neuroblastoma Cells
Source: Front Oncol. 2019 Jul 5;9:579. doi: 10.3389/fonc.2019.00579 (PMC6625372; doi:10.3389/fonc.2019.00579)
Supplement: Supplementary file 3 [file Data_Sheet_3.pdf]

data sheet 3

```
// Crop jpg image according to black square
// CCI - Hendrik Deschout - May 2018

// Get image title and rename if necessary
title = getTitle();
if (endsWith(title, ".tif")) {
        dot_index = indexOf(title, ".tif");
        title = substring (title, 0, dot_index);
        rename(title);
}

// Duplicate image
run("Duplicate...", " ");
title_duplicate = getTitle();

// Convert duplicate to 8-bit and threshold
run("8-bit");
setThreshold(50, 255);
setOption("BlackBackground", true);

// Convert thresholded duplicate to binary and apply binary operations
run("Convert to Mask");
run("Invert");
run("Fill Holes");
erode_repeats = 10;
for (i = 1; i <= erode_repeats; i++) {
        run("Erode");
}

// Determine the ROI of the square region and add to ROI manager
run("Set Measurements...", "bounding limit redirect=None decimal=3");
run("Analyze Particles...", "size=1000-Infinity add");

// Crop original image using ROI
selectWindow(title);
roiManager("Select", 0);
run("Crop");
run("Select None");

// Rename cropped image
rename(title + "_crop");

// Close other images
selectWindow(title_duplicate);
run("Close");
selectWindow("ROI Manager");
run("Close");
```
