# Supplementary figures and images for: Alectinib, an Anaplastic Lymphoma Kinase Inhibitor, Abolishes ALK Activity and Growth in ALK-Positive Neuroblastoma Cells
Source: Front Oncol. 2019 Jul 5;9:579. doi: 10.3389/fonc.2019.00579 (PMC6625372; doi:10.3389/fonc.2019.00579)

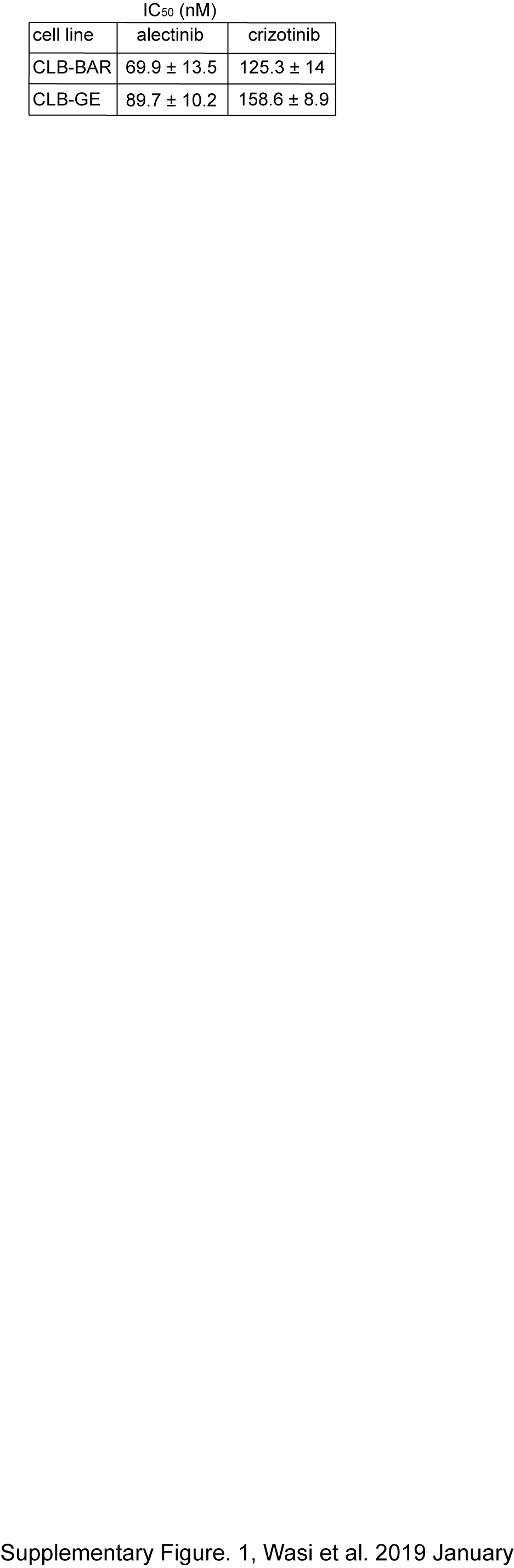

Supplement: Supplementary file 4 [file Image_1.TIF]

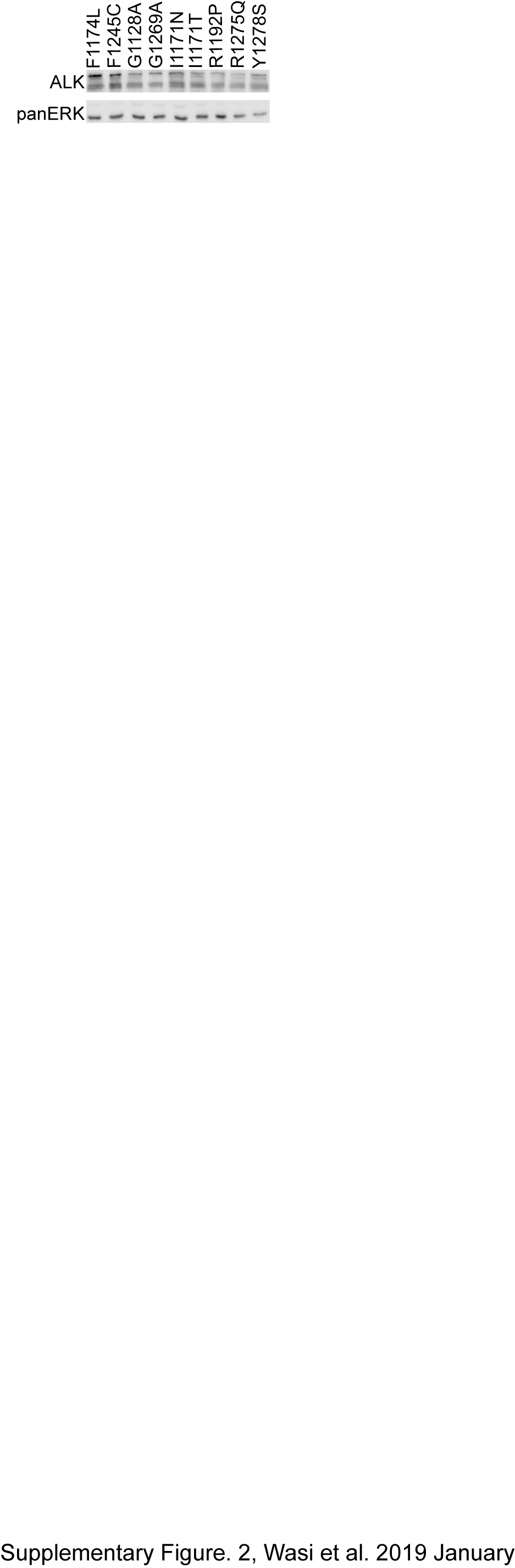

Supplement: Supplementary file 5 [file Image_2.TIF]

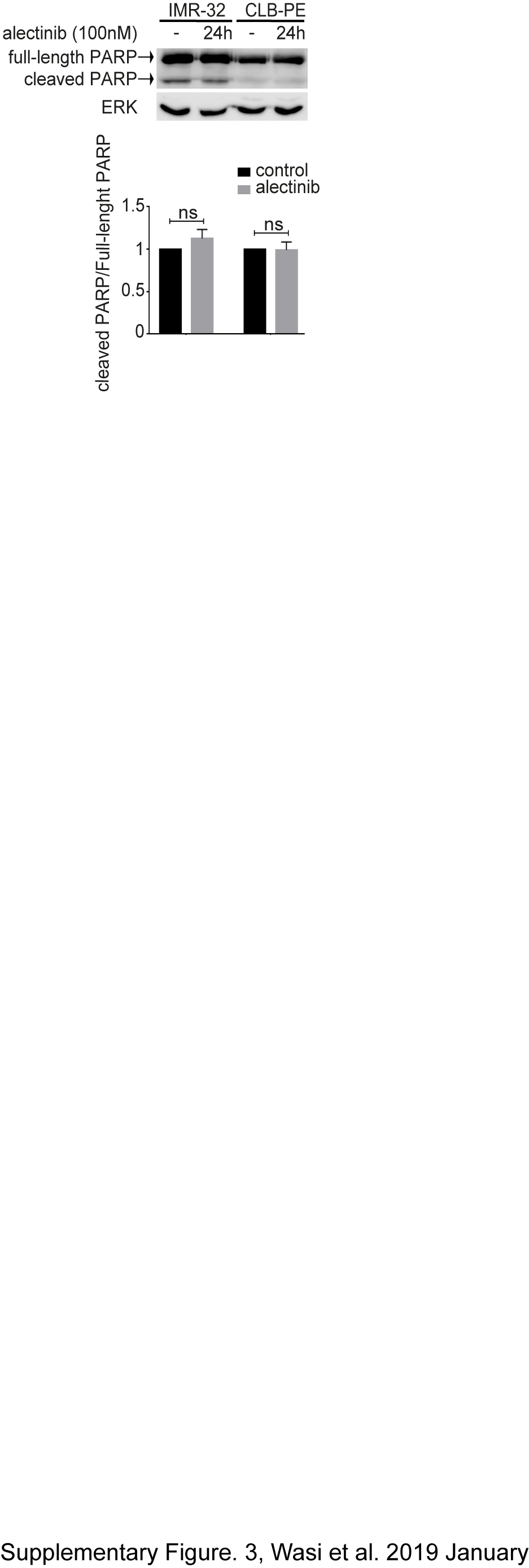

Supplement: Supplementary file 6 [file Image_3.TIF]
